# Baloxavir susceptibility of seasonal influenza viruses during the first seven seasons of clinical use in Japan, 2017/18 to 2023/24

**DOI:** 10.2807/1560-7917.ES.2026.31.1.2500336

**Published:** 2026-01-08

**Authors:** Emi Takashita, Seiichiro Fujisaki, Hiroko Morita, Shiho Nagata, Hideka Miura, Noriko Kishida, Kazuya Nakamura, Masayuki Shirakura, Aya Sato, Miki Akimoto, Hiromi Sugawara, Keiko Mitamura, Takashi Abe, Masataka Ichikawa, Masahiko Yamazaki, Shinji Watanabe, Takato Odagiri, Hideki Hasegawa, the Influenza Virus Surveillance Group of Japan, The Influenza Virus Surveillance Group of Japan, Rika Komagome, Asami Ohnishi, Kyohei Saka, Tomoko Takahashi, Mie Sasaki, Minako Ato, Youko Fujiya, Naomi Ogawa, Nozomi Saito, Miyako Kon, Hiromi Kita, Asaka Okubo, Yuki Wakabayashi, Akira Wakatsuki, Hiroyuki Tsukagoshi, Noriko Hamamoto, Yasuo Kaburagi, Hitomi Yahata, Tomofumi Seno, Mami Nagashima, Sumi Watanabe, Kohei Shimizu, Chie Akaboshi, Yumiko Nagasawa, Ikuyo Kiuchi, Miho Kitazume, Michiko Takeuchi, Erina Miyajima, Asaka Ikegaya, Hirosato Ehara, Ryota Akaike, Takahisa Shimada, Nao Kobashi, Nobushige Sakai, Masahiro Nishioka, Keisuke Futamura, Yoshihiro Yasui, Takuya Yano, Asa Tanino, Yoshihiro Tanabe, Kazuko Nakagawa, Saeko Morikawa, Hiromi Fukuda, Tomohiro Oshibe, Ai Mori, Ryutaro Murayama, Saya Yamamoto, Shiho Nagase, Masaki Nishikawa, Arisa Izumi, Yurie Kanba, Akiyoshi Baba, Yukie Shimazu, Yasutsugu Kawahara, Yayoi Orita, Mizuki Yamamoto, Yukari Tsuchida, Sayako Yoshida, Wataru Satou, Yuri Kondo, Michihiko Miyamoto, Sonoko Izumida, Ukyo Osoegawa, Yumika Takaki, Eisuke Tokuoka, Mayumi Kadoguchi, Toru Hayashi, Nami Tsuru, Yuka Hamada, Noriyuki Maeshiro

**Affiliations:** 1 Influenza Research Center, National Institute of Infectious Diseases, Japan Institute for Health Security, Tokyo, Japan; 2 Eiju General Hospital, Tokyo, Japan; 3 Abe Children’s Clinic, Kanagawa, Japan; 4 Ichikawa Children’s Clinic, Kanagawa, Japan; 5 Zama Children’s Clinic, Kanagawa, Japan; 6 The members of the group are listed under Collaborators.; * These authors contributed equally to this work and share first authorship.

**Keywords:** Influenza, cap-dependent endonuclease inhibitor, baloxavir, resistance

## Abstract

BACKGROUND: Baloxavir marboxil, a cap-dependent endonuclease inhibitor, was approved in Japan in February 2018 for treatment of influenza A and B infections, making Japan the first country to introduce its clinical use.

AIM: We aimed to assess baloxavir susceptibility among seasonal influenza viruses in Japan during the first seven seasons of clinical use, from 2017/18 to 2023/24.

METHODS: We conducted nationwide surveillance on 3,671 influenza viruses using phenotypic and genotypic assays to evaluate baloxavir susceptibility and identify amino acid substitutions in the polymerase acidic (PA) protein associated with reduced susceptibility.

RESULTS: Overall, 1.7% of tested viruses exhibited reduced susceptibility to baloxavir. Influenza A(H3N2) viruses showed the highest frequency (3.6%), followed by influenza A(H1N1)pdm09 (0.9%); no influenza B viruses exhibited reduced susceptibility. Key PA substitutions included E23K, Y24C, I38M/N/S/T/V and E199G/K. Viruses with reduced susceptibility were detected in both treated and untreated individuals. Reduced susceptibility was most frequent during the 2018/19 (4.6%) and 2022/23 (3.2%) seasons, both dominated by A(H3N2) viruses. Notably, the 2018/19 season coincided with peak baloxavir supply to medical institutions, while subsequent seasons with lower antiviral use showed a lower proportion of reduced-susceptibility viruses.

CONCLUSION: Our findings suggest a possible association between the extent of baloxavir use and the emergence of resistance and highlight how circulating subtypes shape seasonal susceptibility profiles. Although reduced susceptibility to baloxavir remains relatively rare, emergence of transmissible virus variants emphasises the need for continued phenotypic and genotypic surveillance to guide treatment strategies, support public health preparedness, and prevent the spread of resistant viruses.

**Key public health message**

**What did you want to address in this study and why?**

We wanted to find out whether seasonal influenza viruses in Japan are becoming less susceptible to baloxavir, an influenza medicine introduced in 2018. If resistance develops, the drug may not work as well for treating patients.

**What have we learnt from this study?**

Only a small proportion of influenza viruses tested showed reduced susceptibility to baloxavir. Resistance appeared more often in some years and in certain virus types, particularly influenza A(H3N2) viruses, especially when baloxavir was widely used. It was also occasionally found in patients who had not taken the drug.

**What are the implications of your findings for public health?**

Although resistance remains uncommon, continued monitoring is crucial. Surveillance ensures that doctors can choose effective treatments, it helps limit the spread of resistant viruses and supports public health planning for future flu seasons.

## Introduction

Baloxavir marboxil, a cap-dependent endonuclease inhibitor, was approved in Japan on 23 February 2018 for the treatment of influenza A and B virus infections, making Japan the first country to authorise its clinical use. On 27 November 2020, its indication was expanded to include prophylactic use for household members or cohabitants of influenza patients who are at high risk of severe disease. For patients aged ≥ 12 years, the approved dose is 80 mg for those weighing > 80 kg, and 40 mg for those weighing ≤ 80 kg. For patients aged < 12 years, the approved therapeutic doses are 40 mg for those weighing ≥ 40 kg, 20 mg for those weighing 20 to < 40 kg, and 10 mg for those weighing 10 to < 20 kg. Prophylactic use is not approved for people in the 10 to < 20 kg weight group.

In Japan, four neuraminidase (NA) inhibitors—oseltamivir, peramivir, zanamivir and laninamivir—and baloxavir are approved for the treatment or prophylaxis of influenza (Table 1). The supply of baloxavir peaked in the 2018/19 season, when a high frequency of treatment-emergent resistance was reported in younger children [1]. This prompted the Japanese Association for Infectious Diseases and the Japan Paediatric Society to issue statements on its use in children aged < 12 years. Although the approval status did not change, these statements may have reduced prescribing in this age group from the 2019/20 season onwards. Since then, excluding the coronavirus disease 2019 (COVID‐19) pandemic period, which impacted the 2020/21 and 2021/22 seasons, baloxavir has had the third-highest supply volume among influenza antivirals, only surpassed by oseltamivir and laninamivir.

**Table 1:** Supply volume of influenza antivirals to medical institutions, Japan, influenza seasons 2017/18–2023/24

Influenza season	Supply volume (unit: 10,000 people)^a^				
	Cap-dependent endonuclease inhibitor	Neuraminidase inhibitor			
	Baloxavir	Oseltamivir	Peramivir	Zanamivir	Laninamivir
2017/18	40	570	60	158	690
2018/19	528	464	32	59	289
2019/20	60	336	19	50	266
2020/21	9.7	8.5	−0.4^b^	0.03	−4.5^b^
2021/22	1.2	1.3	0.8	0	−0.7^b^
2022/23	71.4	203.7	3.4	17.1	90.9
2023/24	365.7	593.2	9.4	68.1	412.8

^a^ Data adapted from the Ministry of Health, Labour, and Welfare, Japan notification on the stable supply of influenza antivirals.

^b^ Negative values indicate that the volume of returns from medical institutions exceeded the supply.

Baloxavir acid, the active form of baloxavir marboxil (hereafter referred to as baloxavir unless otherwise specified), binds to the endonuclease domain of the polymerase acidic (PA) protein and inhibits cleavage of the host pre-mRNA by the PA cap-dependent endonuclease [2]. In Phase 2 and 3 clinical trials of baloxavir, PA substitutions, such as E23K, I38F, I38N and I38T in A(H1N1)pdm09 viruses, E23G/K, A37T, I38M, I38T and E199G in A(H3N2) viruses, and I38T in influenza B viruses, emerged after baloxavir treatment [2-5]. These amino acid substitutions are associated with reduced susceptibility to baloxavir in vitro [5,6], with substitutions at position 38 in the PA protein considered the primary pathway [7]. Of these substitutions, the PA I38T substitution is the most frequently observed and confers the greatest reduction in baloxavir susceptibility in vitro, whereas other I38 substitutions confer smaller reductions in susceptibility [8]. Moreover, in clinical trials, the frequency of PA I38 substitutions in patients infected with influenza A(H3N2) viruses was substantially higher than in those infected with A(H1N1)pdm09 viruses [8]. These studies further showed that among individuals with influenza A(H3N2) infection who received baloxavir, the frequency of PA I38 substitutions was highest in children aged < 6 years (52.2%), followed by those aged 6 to < 12 years (18.9%) and adults aged ≥ 65 years (14.6%) [8]. Baloxavir-treated patients infected with PA I38 mutant viruses exhibit prolonged virus shedding, rebound in virus titres after treatment, and delayed symptom alleviation compared with those infected with wild-type viruses [3,4,6,9].

Since the 2012/13 season, the World Health Organization (WHO) Expert Working Group on Surveillance of Influenza Antiviral Susceptibility (WHO-AVWG) has been monitoring susceptibility to NA inhibitors among globally circulating influenza viruses, with efforts later expanded to include susceptibility to baloxavir after its approval in 2018 [1]. To support public health policy and guide clinical management, we conducted nationwide surveillance of baloxavir susceptibility in circulating influenza viruses in Japan under the WHO-AVWG framework. This report presents findings from the first seven influenza seasons (2017/18 to 2023/24) following the introduction of baloxavir into clinical use.

## Methods

### Viruses

**Figure 1:**
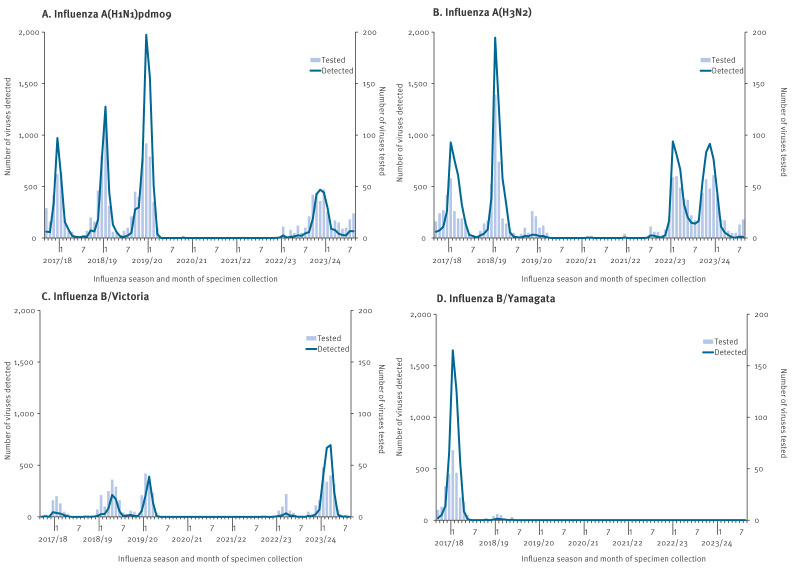
Comparison of influenza virus detections (n = 37,137) and baloxavir susceptibility testing (n = 3,671), Japan, influenza seasons 2017/18–2023/24

Clinical specimens (i.e. throat swabs, sputum, tracheal aspirates, bronchial lavage fluid and bronchoalveolar lavage fluid) along with corresponding patient records, were collected from ca 500 sentinel clinics and hospitals across Japan between the 2017/18 and 2023/24 influenza seasons. In Japan, the influenza surveillance season is defined as spanning from 1 September (week 36) of one year to 31 August (week 35) of the following year, in accordance with the WHO surveillance manual [10]. Despite this year-round framework, seasonal influenza activity in Japan typically peaks during the winter months, most commonly between December and March. Most samples were obtained from untreated patients; however, specimens from individuals undergoing antiviral therapy were also included. During this period, a total of 37,137 seasonal influenza viruses were detected in Japan (WHO FluNet). These included 13,270 A(H1N1)pdm09, 15,592 A(H3N2), 3,801 B/Victoria-lineage and 4,474 B/Yamagata-lineage viruses (Figure 1). Of these, a computer-selected, weighted random subset of 1,378 (10.4%) A(H1N1)pdm09, 1,399 (9.0%) A(H3N2), 608 (16.0%) B/Victoria-lineage and 286 (6.4%) B/Yamagata-lineage viruses was tested for baloxavir susceptibility using a combination of phenotypic and genotypic analyses. The weighting prioritised viruses from a region or subtype not yet sampled in the same season, or for which sufficient time had elapsed since the previous sampling, with ca 10% of reported viruses selected each week for testing.

Monthly reports of virus detection are based on data from the World Health Organization FluNet (https://www.who.int/tools/flunet).

### Phenotypic analysis using the focus reduction assay

Baloxavir susceptibility was determined phenotypically by using a focus reduction assay as previously described [11]. We infected confluent monolayers of cells in 96-well plates with 1,000 focus-forming units of virus per well. After 1 h of adsorption at 37 °C, an equal volume of Avicel RC-581 (DuPont Nutrition, United States) in culture medium containing serial dilutions of baloxavir acid and TPCK-trypsin was added to each well. The cells were then incubated for 18 h at 34 °C, fixed with formalin, immunostained with mouse monoclonal antibodies against influenza nucleoprotein, and detected with horseradish peroxidase-labelled goat anti-mouse immunoglobulin (SeraCare Life Sciences, United States). Infected foci were visualised with TrueBlue Substrate (SeraCare Life Sciences) and quantified using an ImmunoSpot S6 Analyzer with ImmunoCapture and BioSpot software (Cellular Technology, United States). We tested each virus in triplicate. In the 2017/18 season, influenza A(H1N1)pdm09 and B viruses were tested using MDCK cells, whereas A(H3N2) viruses were tested using a combination of MDCK cells and two types of modified MDCK cells: MDCK-SIAT1 cells, which overexpress α2,6-sialyltransferase [12], and humanised MDCK cells (hCK cells), which predominantly express α2,6-linked sialoglycans with very low expression of α2,3-linked sialoglycans [13]. In the 2018/19 season, influenza A(H1N1)pdm09 and B viruses continued to be tested using MDCK cells, whereas A(H3N2) viruses were tested using hCK cells because they grew best in these cells in that season. Since the 2019/20 season, hCK cells have been used to test all virus types. Baloxavir acid was purchased from Funakoshi Co., Ltd. (Japan). The results are expressed as 50% effective concentration (EC_50_) values, calculated using GraphPad Prism (GraphPad Software, United States). To interpret baloxavir susceptibility, we applied the provisional criteria proposed by the WHO-AVWG, using EC_50_ fold-change values relative to the median values for viruses from the same type/subtype/lineage, as previously described [1]. Specifically, we calculated the median EC_50_ values from viruses collected in Japan during the same influenza season. The provisional WHO-AVWG criteria define susceptibility as normal (≤ 3-fold increase) or reduced (> 3-fold increase).

### Genotypic analysis by sequencing

Genotypic analysis was conducted using Sanger sequencing and next-generation sequencing (NGS) to detect amino acid substitutions associated with reduced baloxavir susceptibility. A list of such substitutions has been published by the WHO-AVWG [14]. Sanger sequencing was performed as previously described [15]. We amplified full-length PA gene segments from viral RNAs using the SuperScript III One-step RT-PCR system with Platinum Taq (Thermo Fisher Scientific, United States), followed by purification with AMPure XP beads (Beckman Coulter, United States). Nucleotide sequences were determined using the BigDye terminator v3.1 cycle sequencing kit (Thermo Fisher Scientific) and analysed with the Applied Biosystems 3730xl DNA Analyzer (Thermo Fisher Scientific). We performed NGS as previously described [16]. A cDNA library was prepared using the QIAseq FX DNA Library Kit (Qiagen, Germany), followed by purification with AMPure XP beads (Beckman Coulter). Sequencing was conducted using the MiSeq Reagent Kit v3 or the iSeq 100 i1 Reagent Kit with MiSeq or iSeq 100 instruments, respectively (Illumina, United States). Sequence reads were aligned to reference sequences using the QIAGEN CLC Genomics Workbench (Qiagen). These reference sequences corresponded to the influenza vaccine strains recommended by the WHO for each influenza season. All sequence data are available in the EpiFlu database of the Global Initiative on Sharing All Influenza Data (GISAID). Amino acid position numbering is consistent across influenza A and B viruses.

## Results

### Detection of seasonal influenza viruses with reduced susceptibility to baloxavir in Japan in the 2017/18–2023/24 influenza seasons

**Figure 2:**
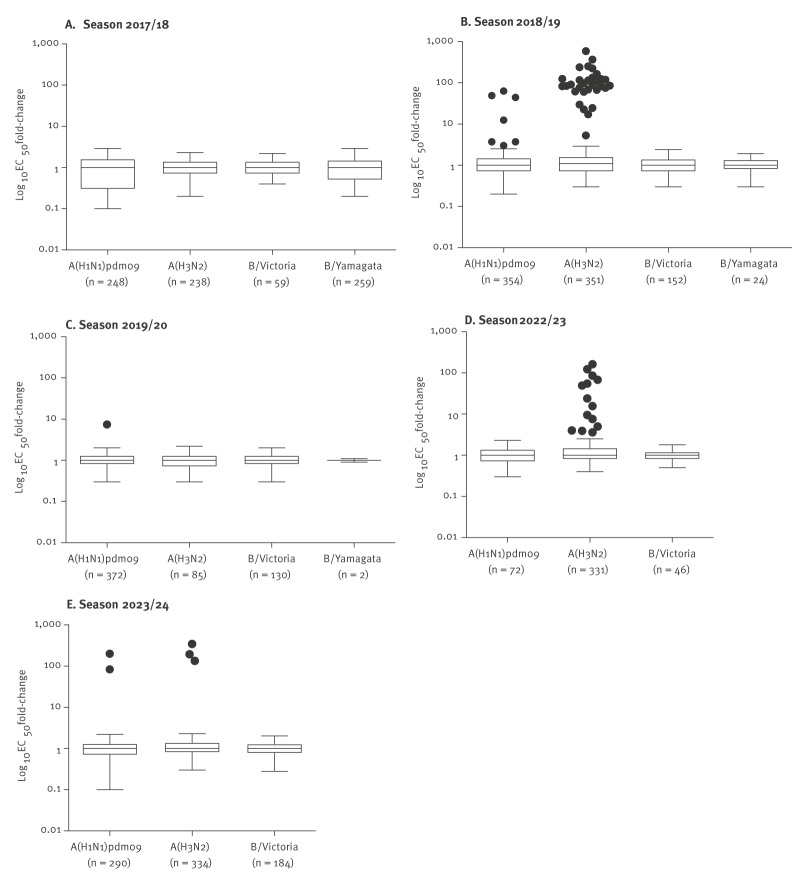
Baloxavir susceptibility of seasonal influenza viruses collected, Japan, influenza seasons 2017/18–2023/24 (n = 3,558)

To monitor the baloxavir susceptibility of circulating influenza viruses in Japan, we examined seasonal influenza viruses collected between the 2017/18 and 2023/24 seasons. Consistent with influenza activity levels, we tested 1,378 A(H1N1)pdm09, 1,399 A(H3N2), 608 B/Victoria-lineage, and 286 B/Yamagata-lineage viruses (Figure 1). The majority of viruses were evaluated using phenotypic and genotypic assays. However, for viruses with low titres that could not be assessed with the cell-based phenotypic assay (i.e. 39 A(H1N1)pdm09, 37 A(H3N2), 36 B/Victoria-lineage and one B/Yamagata-lineage viruses), only the genotypic assay was performed. Fold-change values in EC_50_, calculated relative to the subtype- or lineage-specific median values obtained with the respective cell lines, are shown in Figure 2. The corresponding median EC_50_ values by subtype/lineage and season are summarised in Supplementary Table S1. Overall, influenza B viruses showed higher median EC_50_ values than influenza A viruses, and B/Yamagata-lineage viruses were last detected in the 2019/20 season.

EC_50_: 50% effective concentration.

The EC_50_ values of the viruses to baloxavir were determined in a focus reduction assay. Box-and-whisker plots show the fold-change in EC_50_ values relative to the median for viruses of the same type, subtype, or lineage; boxes indicate the interquartile range, and horizontal lines indicate the median. Black circles indicate viruses with reduced susceptibility to baloxavir. Data for the 2020/21 and 2021/22 seasons are not shown because of the very limited number of viruses tested.

The frequency of seasonal influenza viruses exhibiting reduced susceptibility to baloxavir is shown in Table 2, and additional details on these viruses are appended in Supplementary Table S2.

**Table 2:** Frequency of seasonal influenza viruses exhibiting reduced susceptibility to baloxavir in Japan, influenza seasons 2017/18–2023/24 (n = 3,671)

Influenza season	Total frequency			Type/subtype/lineage											
				A(H1N1)pdm09			A(H3N2)			B/Victoria			B/Yamagata		
	%	n	Total	%	n	Total	%	n	Total	%	n	Total	%	n	Total
2017/18	0	0	833	0	0	257	0	0	256	0	0	60	0	0	260
2018/19	4.6	41	900	2.2	8	367	9.3	33	356	0	0	153	0	0	24
2019/20	0.2	1	612	0.3	1	378	0	0	89	0	0	143	0	0	2
2020/21	0	0	6	0	0	2	0	0	4	0	0	0	0	0	0
2021/22	0	0	23	0	0	2	0	0	21	0	0	0	0	0	0
2022/23	3.2	15	465	1.3	1	75	4.1	14	339	0	0	51	0	0	0
2023/24	0.6	5	832	0.7	2	297	0.9	3	334	0	0	201	0	0	0
**Total**	**1.7**	**62**	**3,671**	**0.9**	**12**	**1,378**	**3.6**	**50**	**1,399**	**0**	**0**	**608**	**0**	**0**	**286**

During the 2017/18 season, baloxavir supply volume was the lowest among anti-influenza drugs (Table 1), indicating minimal clinical use. A total of 10,228 viruses were detected, with influenza B/Yamagata-lineage viruses the most frequently identified (43%, n = 4,424), followed by A(H3N2) (33%, n = 3,321), A(H1N1)pdm09 (23%, n = 2,339) and B/Victoria-lineage viruses (1%, n = 144) (Figure 1). No viruses with reduced susceptibility to baloxavir were detected during this period (Table 2).

During the 2018/19 season, baloxavir was the most widely supplied anti-influenza drug, with 5.28 million treatment courses distributed (Table 1). A total of 8,482 viruses were detected, with A(H3N2) viruses predominating (56%, n = 4,742), followed by A(H1N1)pdm09 (36%, n = 3,088), B/Victoria-lineage viruses (7%, n = 606) and B/Yamagata-lineage viruses (1%, n = 46) (Figure 1). Viruses with reduced susceptibility to baloxavir were identified in 2.2% (8/367) of A(H1N1)pdm09 viruses and 9.3% (33/356) of A(H3N2) viruses (Table 2). Among A(H1N1)pdm09 viruses, six were from children aged < 12 years, and three possessed a PA I38V substitution detected in patients who had not received baloxavir before specimen collection (Table 3). These viruses exhibited 3.0–3.7-fold higher EC_50_ values against baloxavir compared with viruses without known PA substitutions. In contrast, A(H1N1)pdm09 viruses with the PA I38S or I38T substitution showed markedly elevated EC_50_ values. We also detected A(H1N1)pdm09 viruses harbouring the mixed substitutions PA I38F/I and I38F/T. Among A(H3N2) viruses, 26 were from children aged < 12 years, and five were from untreated patients carrying a PA I38T substitution (Table 3). In total, 22 A(H3N2) viruses possessed the PA I38T substitution; 21 showed markedly elevated EC_50_ values, while one could not be phenotypically tested because its viral titre was too low for recovery. Additional substitutions included the PA I38M, I38M/I, I38M/R/I, I38M/T/I and I38T/I.

**Table 3:** Characteristics of influenza A viruses exhibiting reduced susceptibility to baloxavir in Japan, influenza seasons 2017/18–2023/24 (n = 62)

PA substitution	EC_50_ fold-change^a^	Baloxavir treatment^b^	Number of patients
Influenza A(H1N1)pdm09			
E23K	7.4	No	1
I38F/I mix (F: 40%)	2.5	Yes	1
I38F/T mix (F: 41%; T: 59%)	12.5	Yes	1
I38N	83.3	No	1
I38S	49.1	Yes	1
I38T	199.4^c^	No	2
	44.2–63.3	Yes	2
I38V	3.0–3.7	No	3
Influenza A(H3N2)			
Y24C	3.9–9.5	No	2
I38M	15.6–22.7	Yes	2
I38M/I mix (M: 62%)	24.5	Yes	1
I38M/R/I mix (M: 18%; R: 18%)	2.0	Yes	1
I38M/T/I mix (M: 21–74%; T: 14–40%)	24.0–88.0	Yes	2
I38T	67.9–368.0	No	7
	55.0–588.7	Yes	22
I38T/I mix (T: 16–88%)	5.3–239.4	Yes	9
E199G	3.6–7.6	No	3
E199K	5.0	No	1

PA: polymerase acidic subunit; EC_50_: 50% effective concentration.

^a^ Fold-change in EC_50_ values for baloxavir compared with the median EC_50_ values of influenza A(H1N1)pdm09 and A(H3N2) viruses collected in Japan during the same influenza season.

^b^ Treatment with baloxavir before specimen collection.

^C^ One virus was not phenotypically tested due to low viral titre.

In the 2019/20 season, the Japan Paediatric Society and the Japanese Association for Infectious Diseases advised against the routine use of baloxavir in children under 12 years and discouraged monotherapy in severely immunosuppressed patients [8]. Consequently, baloxavir distribution dropped to 0.6 million treatment courses, ranking it third among anti-influenza drugs that season (Table 1). A total of 6,398 viruses were detected, with A(H1N1)pdm09 viruses predominating (85%, n = 5,419), followed by B/Victoria-lineage (13%, n = 834), A(H3N2) (2%, n = 141) and B/Yamagata-lineage viruses (0.1%, n = 4) (Figure 1). We detected one A(H1N1)pdm09 virus carrying a PA E23K substitution in a patient with no prior baloxavir exposure (Table 3), representing 0.3% (1/378) of the tested A(H1N1)pdm09 viruses (Table 2). This virus exhibited a 7.4-fold increase in EC_50_ value (Table 3).

Influenza activity was minimal during the 2020/21 and 2021/22 seasons because of the COVID-19 pandemic, with only a few influenza A viruses and no influenza B viruses detected (Figure 1). Correspondingly, the supply volumes of all anti-influenza drugs declined (Table 1), and no viruses with reduced susceptibility to baloxavir were detected during these seasons (Table 2). Since the 2020/21 season, no B/Yamagata-lineage viruses have been documented.

Influenza activity re-emerged in the 2022/23 season. A total of 3,909 viruses were detected, with A(H3N2) viruses predominating (92%, n = 3,610), far exceeding the number of A(H1N1)pdm09 (5%, n = 212) and B/Victoria-lineage viruses (2%, n = 87) (Figure 1). Viruses with reduced susceptibility to baloxavir were detected in 1.3% (1/75) of A(H1N1)pdm09 viruses and 4.1% (14/339) of A(H3N2) viruses (Table 2). The A(H1N1)pdm09 virus possessing a PA I38T substitution was detected in an untreated patient but could not be phenotypically tested because its viral titre was too low for recovery. Among the 14 influenza A(H3N2) viruses, eight were from untreated patients and carried the PA substitution Y24C, I38T, E199G or E199K (Table 3). We also detected influenza A(H3N2) viruses carrying the PA I38M, I38M/T/I, and I38T/I substitutions. Two A(H3N2) viruses carrying the PA Y24C substitution exhibited 3.9- and 9.5-fold increases in EC_50_ values (Table 3). Compared with their most closely related wild-type viruses, the PA Y24C viruses exhibited 2.0–2.1-fold and 4.9–5.3-fold higher EC_50_ values. Supplementary Table S3 provides additional data on the amino acid differences between these viruses and their wild-type counterparts, indicating only a single substitution in haemagglutinin, which has not been associated with altered antiviral susceptibility or viral replication.

In the 2023/24 season, a total of 8,061 viruses were detected. Influenza A(H3N2) viruses again predominated (46%, n = 3,723), but A(H1N1)pdm09 (27%, n = 2,208) and B/Victoria-lineage viruses (26%, n = 2,130) were detected more frequently than in the previous season (Figure 1). Viruses with reduced susceptibility to baloxavir were detected in 0.7% (2/297) of A(H1N1)pdm09 viruses and 0.9% (3/334) of A(H3N2) viruses (Table 2). The A(H1N1)pdm09 viruses were from untreated patients and possessed the PA I38T or I38N substitutions [17], both associated with markedly elevated EC_50_ values (Table 3). Among A(H3N2) viruses, the PA I38T and I38T/I substitutions were identified; all were from baloxavir-treated patients and were associated with elevated EC_50_ values (Table 3).

### Age group distribution of seasonal influenza viruses with reduced susceptibility to baloxavir in Japan in the 2017/18–2023/24 influenza seasons

The age-specific distribution of viruses with reduced susceptibility to baloxavir is presented in Table 4.

**Table 4:** Distribution by patient age group of seasonal influenza viruses exhibiting reduced susceptibility to baloxavir, Japan, 2017/18–2023/24 influenza seasons (n = 3,671)

Age group (years)	Total frequency			Type/subtype/lineage											
				A(H1N1)pdm09			A(H3N2)			B/Victoria			B/Yamagata		
	%	n	Total	%	n	Total	%	n	Total	%	n	Total	%	n	Total
0–5	1.5	13	881	0.6	2	355	3.2	11	349	0	0	112	0	0	65
6–11	2.0	26	1,319	1.3	7	560	4.4	19	427	0	0	244	0	0	88
12–64	1.5	20	1,314	0.7	3	410	3.1	17	551	0	0	241	0	0	112
≥ 65	2.2	3	138	0	0	45	4.4	3	68	0	0	5	0	0	20
Unknown	0	0	19	0	0	8	0	0	4	0	0	6	0	0	1

All influenza viruses with reduced susceptibility were either A(H1N1)pdm09 or A(H3N2) viruses. Across all age groups, the frequency of viruses with reduced susceptibility was consistently higher among patients infected with A(H3N2) viruses than among those infected with A(H1N1)pdm09 viruses, in line with findings from the clinical trials of baloxavir [8]. We detected A(H1N1)pdm09 viruses with reduced susceptibility in all age groups except the group ≥ 65 years, with the highest frequency in children aged 6–11 years (1.3%, 7/560), followed by those aged 12–64 years (0.7%, 3/410) and 0–5 years (0.6%, 2/355). Among A(H3N2) viruses, the highest frequencies occurred in the 6–11-year and ≥ 65-year groups (both 4.4%, 19/427 and 3/68, respectively), followed by children aged 0–5 years (3.2%, 11/349) and people aged 12–64 years (3.1%, 17/551). No B/Victoria- or B/Yamagata-lineage viruses with reduced baloxavir susceptibility were detected in any age group.

## Discussion

This study provides a comprehensive phenotypic and genotypic analysis of baloxavir susceptibility among seasonal influenza viruses collected in Japan from the 2017/18 to 2023/24 seasons. Japan, the first country to approve baloxavir for clinical use, has remained its largest market, offering a unique opportunity to monitor the emergence and evolution of resistance at the population level through nationwide surveillance.

During the study period, 1.7% of tested influenza viruses showed reduced susceptibility to baloxavir. The highest frequency was observed among A(H3N2) viruses, followed by A(H1N1)pdm09 viruses. The highest rates of reduced susceptibility occurred in the 2018/19 and 2022/23 seasons, both dominated by A(H3N2) viruses, a subtype consistently associated with a higher likelihood of resistance. These observations indicate that influenza A(H3N2) predominance substantially contributes to the seasonal burden of antiviral resistance. Notably, fitness deficits appeared more pronounced in A(H1N1)pdm09 viruses than in A(H3N2) viruses [18], which may help explain the lower frequency of resistant A(H1N1)pdm09 variants in clinical settings despite the recurrent detection of resistance in both subtypes. Influenza A(H3N2) viruses may therefore be better able to sustain transmission of resistant variants, highlighting the importance of subtype-specific surveillance.

The 2018/19 season also coincided with the highest baloxavir supply, suggesting a link between increased baloxavir use and the emergence of resistance. In contrast, following the issuance of clinical guidance in 2019 discouraging baloxavir use in certain paediatric and immunocompromised populations, the national supply volume declined markedly, accompanied by a decrease in the detection of viruses with reduced susceptibility in the 2019/20 season. Together, these trends support a possible association between the extent of baloxavir use and the emergence of resistance, while highlighting how circulating subtypes shape seasonal susceptibility profiles.

Our age-stratified analysis revealed that the highest frequency of viruses with reduced susceptibility occurred in individuals aged ≥ 65 years and children aged 6–11 years, followed by those aged 0–5 years and 12–64 years. Among the three older adults with viruses with reduced susceptibility, one patient had received baloxavir treatment before specimen collection, whereas the other two cases were sporadic detections without baloxavir exposure; all three cases involved A(H3N2) viruses. Although the sample size is small, these findings suggest the presence of both treatment-emergent and community-acquired resistance. These results further indicate that older adults, as well as children, may be prone to harbouring or acquiring A(H3N2) viruses with reduced baloxavir susceptibility, potentially owing to prolonged viral replication or underlying health conditions. Notably, unlike oseltamivir, which is widely used in children and available as a paediatric-friendly oral suspension, baloxavir is not available as a liquid formulation suitable for young children in Japan. Although baloxavir granules for oral suspension were approved in Japan in September 2018, they were not launched until 12 November 2025, which was after the period covered in this study. Even after their launch, prophylactic use remains unapproved for people in the < 20 kg weight group. This difference in formulation availability may partly explain the limited use of baloxavir in children aged 0–5 years and the correspondingly lower frequency of influenza viruses with reduced susceptibility detection in this age group compared with children aged 6–11 years.

During this study, we identified a novel PA Y24C substitution. Structural studies indicate that baloxavir acid interacts with the A20, Y24, K34, A37, and I38 residues in the influenza A virus PA protein [2]. Substitution of Y24 with a smaller residue such as cysteine may therefore disrupt binding and reduce baloxavir affinity. This finding highlights the importance of continued monitoring for emerging PA mutations, especially those with unknown effects.

The PA I38T, E199G and E199K substitutions are known to confer reduced susceptibility to baloxavir [14,19,20]. The PA E199G mutant viruses were associated with a community cluster [19]. The PA I38V substitution slightly shortens the hydrophobic side chain and is therefore unlikely to cause major structural alterations affecting baloxavir susceptibility. Consistent with this, previous work reported only a twofold increase in EC_50_ value for an A(H1N1)pdm09 virus with the PA I38V substitution compared with a sequence-matched control [21]. 

Globally, seasonal influenza viruses with reduced susceptibility to baloxavir remain rare. According to the most recent WHO-AVWG global update for the 2022/23 season, 0.05–0.12% of viruses carried PA substitutions associated with reduced baloxavir susceptibility, most frequently I38T/M/L, with detections across Europe, North America and the Western Pacific [22]. Japan has reported higher frequencies than other regions, probably reflecting both greater baloxavir use and extensive phenotypic testing capacity. Despite these differences, resistant variants remain uncommon globally, underscoring the importance of continued international surveillance.

Importantly, some viruses with reduced susceptibility were detected in patients without prior baloxavir exposure, indicating possible human-to-human transmission. Consistent with this observation, A(H1N1)pdm09 and A(H3N2) viruses carrying the PA I38T substitution, isolated from baloxavir-treated patients in Japan, show comparable replication fitness and pathogenicity to wild-type viruses in hamster models and can efficiently transmit between ferrets via respiratory droplets [23]. Additional ferret studies confirmed that viruses with the PA E23G or E23K substitution also retain transmissibility with minimal fitness loss [18].

In interpreting the EC_50_ data, it is important to consider differences in the cell systems used across seasons. Because A(H3N2) viruses replicated more efficiently in hCK cells than in MDCK or MDCK-SIAT1 cells, the corresponding EC_50_ values were higher in hCK cells. This difference should be considered when comparing across cell systems, although fold-change interpretations within the same cell system remain valid. The observed fold-changes are likely to reflect viral genetic backgrounds and assay variability.

Limitations of this study include variability in the proportion of specimens obtained after antiviral treatment, which may have influenced resistance detection. In addition, following reports of treatment-emergent resistance in younger children during the 2018/19 season, professional society statements may have reduced baloxavir use in those aged < 12 years, potentially affecting resistance frequencies. Finally, supply volume in Japan is used as a surrogate for prescription trends but does not fully reflect the number of patients treated.

Although the frequency of viruses with reduced susceptibility remains relatively low, the emergence of transmissible variants, particularly in the absence of selective drug pressure, highlights the need for continued vigilance. Continuous monitoring of baloxavir susceptibility is essential, especially as its use persists and influenza virus circulation returns to pre-pandemic levels. Given the clinical consequences of resistance, including delayed symptom resolution, prolonged viral shedding and virological rebound, early detection of resistant variants through integrated virological and clinical surveillance is essential to inform antiviral policy, guide treatment strategies and prevent the spread of resistant viruses. These observations also highlight the broader public health importance of maintaining coordinated global surveillance, particularly as influenza circulation and international travel continue to normalise.

## Conclusion

This seven-season analysis of baloxavir susceptibility in Japan shows that viruses with reduced susceptibility remain uncommon, but transmissible variants are present. These findings underscore the importance of integrating antiviral susceptibility data into national surveillance and treatment guidelines to mitigate the risk of resistant influenza spread.

## Data Availability

All sequence data are available in the EpiFlu database of the Global Initiative on Sharing All Influenza Data (GISAID).

## Supplementary Data

293000 Supplement

## References

[1] Govorkova EA Takashita E Daniels RS Fujisaki S Presser LD Patel MC Global update on the susceptibilities of human influenza viruses to neuraminidase inhibitors and the cap-dependent endonuclease inhibitor baloxavir, 2018-2020. Antiviral Res. 2022;200:105281. 35292289 10.1016/j.antiviral.2022.105281 PMC9254721

[2] Omoto S Speranzini V Hashimoto T Noshi T Yamaguchi H Kawai M Characterization of influenza virus variants induced by treatment with the endonuclease inhibitor baloxavir marboxil. Sci Rep. 2018;8(1):9633. 29941893 10.1038/s41598-018-27890-4 PMC6018108

[3] Hayden FG Sugaya N Hirotsu N Lee N de Jong MD Hurt AC Baloxavir marboxil for uncomplicated influenza in adults and adolescents. N Engl J Med. 2018;379(10):913-23. 30184455 10.1056/NEJMoa1716197

[4] Hirotsu N Sakaguchi H Sato C Ishibashi T Baba K Omoto S Baloxavir marboxil in Japanese pediatric patients with influenza: safety and clinical and virologic outcomes. Clin Infect Dis. 2020;71(4):971-81. 31538644 10.1093/cid/ciz908 PMC7428393

[5] Ince WL Smith FB O’Rear JJ Thomson M. Treatment-emergent influenza virus polymerase acidic substitutions independent of those at i38 associated with reduced baloxavir susceptibility and virus rebound in trials of baloxavir marboxil. J Infect Dis. 2020;222(6):957-61. 32253432 10.1093/infdis/jiaa164

[6] Uehara T Hayden FG Kawaguchi K Omoto S Hurt AC De Jong MD Treatment-emergent influenza variant viruses with reduced baloxavir susceptibility: impact on clinical and virologic outcomes in uncomplicated influenza. J Infect Dis. 2020;221(3):346-55. 31309975 10.1093/infdis/jiz244

[7] Ison MG Hayden FG Hay AJ Gubareva LV Govorkova EA Takashita E Influenza polymerase inhibitor resistance: Assessment of the current state of the art - A report of the isirv Antiviral group. Antiviral Res. 2021;194:105158. 34363859 10.1016/j.antiviral.2021.105158 PMC9012257

[8] Takashita E. Influenza polymerase inhibitors: mechanisms of action and resistance. Cold Spring Harb Perspect Med. 2021;11(5):a038687. 32122918 10.1101/cshperspect.a038687 PMC8091960

[9] Sato M Takashita E Katayose M Nemoto K Sakai N Fujisaki S Detection of variants with reduced baloxavir marboxil and oseltamivir susceptibility in children with influenza a during the 2019-2020 influenza season. J Infect Dis. 2021;224(10):1735-41. 33837427 10.1093/infdis/jiab196

[10] World Health Organization (WHO). Global epidemiological surveillance standards for influenza. Geneva: WHO; 2013. Available from: https://www.who.int/publications/i/item/9789241506601

[11] Takashita E Morita H Ogawa R Nakamura K Fujisaki S Shirakura M Susceptibility of influenza viruses to the novel cap-dependent endonuclease inhibitor baloxavir marboxil. Front Microbiol. 2018;9:3026. 30574137 10.3389/fmicb.2018.03026 PMC6291754

[12] Matrosovich M Matrosovich T Carr J Roberts NA Klenk HD. Overexpression of the alpha-2,6-sialyltransferase in MDCK cells increases influenza virus sensitivity to neuraminidase inhibitors. J Virol. 2003;77(15):8418-25. 12857911 10.1128/JVI.77.15.8418-8425.2003 PMC165236

[13] Takada K Kawakami C Fan S Chiba S Zhong G Gu C A humanized MDCK cell line for the efficient isolation and propagation of human influenza viruses. Nat Microbiol. 2019;4(8):1268-73. 31036910 10.1038/s41564-019-0433-6 PMC12421904

[14] World Health Organization (WHO). Summary of polymerase acidic (PA) protein amino acid substitutions analysed for their effects on baloxavir susceptibility. Geneva: WHO; 2024. Available from: https://www.who.int/publications/m/item/summary-of-polymerase-acidic-(pa)-protein-amino-acid-substitutions-analysed-for-their-effects-on-baloxavir-susceptibility

[15] Takashita E Ejima M Ogawa R Fujisaki S Neumann G Furuta Y Antiviral susceptibility of influenza viruses isolated from patients pre- and post-administration of favipiravir. Antiviral Res. 2016;132:170-7. 27321665 10.1016/j.antiviral.2016.06.007

[16] Shimizu K Kawakami C Matsuzaki Y Fujisaki S Nagata S Morita H Monitoring influenza C and D viruses in patients with respiratory diseases in Japan, January 2018 to March 2023. Influenza Other Respir Viruses. 2024;18(6):e13345. 38923307 10.1111/irv.13345 PMC11196370

[17] Takashita E Morita H Nagata S Fujisaki S Miura H Ikeda T Influenza A(H1N1)pdm09 virus with reduced susceptibility to Baloxavir, Japan, 2024. Emerg Infect Dis. 2025;31(5):1019-23. 40180581 10.3201/eid3105.241123 PMC12044245

[18] Jones JC Zagribelnyy B Pascua PNQ Bezrukov DS Barman S Okda F Influenza A virus polymerase acidic protein E23G/K substitutions weaken key baloxavir drug-binding contacts with minimal impact on replication and transmission. PLoS Pathog. 2022;18(7):e1010698. 35830486 10.1371/journal.ppat.1010698 PMC9312377

[19] Takashita E Fujisaki S Morita H Nagata S Miura H Matsuura Y A community cluster of influenza A(H3N2) virus infection with reduced susceptibility to baloxavir due to a PA E199G substitution in Japan, February to March 2023. Euro Surveill. 2023;28(39):2300501. 37768560 10.2807/1560-7917.ES.2023.28.39.2300501 PMC10540515

[20] Takashita E Yasui Y Ikegaya A Saka K Maeshiro N Morita H Impact of the polymerase acidic protein E199K substitution in influenza A viruses on baloxavir susceptibility. Antiviral Res. 2025;239:106173. 40324596 10.1016/j.antiviral.2025.106173

[21] Gubareva LV Mishin VP Patel MC Chesnokov A Nguyen HT De La Cruz J Assessing baloxavir susceptibility of influenza viruses circulating in the United States during the 2016/17 and 2017/18 seasons. Euro Surveill. 2019;24(3):1800666. 30670144 10.2807/1560-7917.ES.2019.24.3.1800666 PMC6344838

[22] Hussain S Meijer A Govorkova EA Dapat C Gubareva LV Barr IG Global update on the susceptibilities of influenza viruses to neuraminidase inhibitors and the cap-dependent endonuclease inhibitor baloxavir, 2020-2023. Antiviral Res. 2025;241:106217. 40571063 10.1016/j.antiviral.2025.106217 PMC12391581

[23] Imai M Yamashita M Sakai-Tagawa Y Iwatsuki-Horimoto K Kiso M Murakami J Influenza A variants with reduced susceptibility to baloxavir isolated from Japanese patients are fit and transmit through respiratory droplets. Nat Microbiol. 2020;5(1):27-33. 31768027 10.1038/s41564-019-0609-0 PMC13014278

