# Supplementary material for: Baloxavir susceptibility of seasonal influenza viruses during the first seven seasons of clinical use in Japan, 2017/18 to 2023/24
Source: Euro Surveill. 2026 Jan 8;31(1):2500336. doi: 10.2807/1560-7917.ES.2026.31.1.2500336 (PMC12862291; doi:10.2807/1560-7917.ES.2026.31.1.2500336)
Supplement: Supplement [file 25-00336_TAKASHITA_Supplement.pdf]

Supplement

This supplementary material is hosted by *Eurosurveillance* as supporting information alongside the article "Baloxavir susceptibility of seasonal influenza viruses during the first seven seasons of clinical use in Japan, 2017/18 to 2023/24", on behalf of the authors, who remain responsible for the accuracy and appropriateness of the content. The same standards for ethics, copyright, attributions and permissions as for the article apply. Supplements are not edited by *Eurosurveillance* and the journal is not responsible for the maintenance of any links or email addresses provided therein.

Supplementary Table 1. Median EC_{50} values for baloxavir susceptibility of seasonal influenza viruses collected in Japan, 2017/18–2023/24 influenza seasons

Influenza season	Type/subtype/lineage (Median EC_{50}, nM)			
	A(H1N1)pdm09	A(H3N2)	B/Victoria	B/Yamagata
2017/18	1.08	0.15; 0.20; 2.31	7.83	13.79
2018/19	1.49	1.91	20.51	19.76
2019/20	5.61	3.43	28.81	33.51
2020/21	ND	3.19	NA	NA
2021/22	ND	3.59	NA	NA
2022/23	2.98	4.15	49.28	NA
2023/24	4.65	4.70	47.59	NA

EC_{50}: 50% effective concentration; ND: not determined (very limited number of viruses); NA: not applicable (no virus detected).

The EC_{50} values of the viruses to baloxavir were determined by use of a focus reduction assay. In 2017/18, A(H1N1)pdm09 and B viruses were tested using MDCK cells, whereas A(H3N2) viruses were tested using three different cell systems (MDCK, MDCK-SIAT1, and hCK); the EC_{50} values in the table correspond to these systems. In 2018/19, A(H1N1)pdm09 and B viruses were tested using MDCK cells, and A(H3N2) viruses using hCK cells. Since 2019/20, hCK cells have been used for all virus types. Numbers of viruses tested are shown in Figure 2.

Supplementary Table 2. Characteristics of patients and influenza A viruses exhibiting reduced susceptibility to baloxavir in Japan, 2017/18–2023/24 influenza seasons (n=62)

Influenza season	Subtype	Isolate name	PA substitution	EC_{50} fold-change[a]	Baloxavir treatment[b]	Collection date	GISAID Isolate ID	Age (years)
2018/19	A(H1N1)pdm09	A/Wakayama/21/2019	I38F/I mix (F: 40%)	2.5	Yes	2019-02-04	803868	9
		A/Kanagawa/88/2018	I38F/T mix (F: 41%; T: 59%)	12.5	Yes	2018-11-20	337454	8
		A/Yokohama/98/2019	I38S	49.1	Yes	2019-02-08	356745	6
		A/Kanagawa/AC1830/2019	I38T	63.3	Yes	2019-01-15	354782	14
		A/Kanagawa/IC1890/2019	I38T	44.2	Yes	2019-01-28	416540	9
		A/Ibaraki/37/2018	I38V	3.7	No	2018-11-07	398306	7
		A/Ibaraki/38/2018	I38V	3.7	No	2018-11-07	398307	6
		A/Ibaraki/42/2018	I38V	3.0	No	2018-11-27	398308	38
	A(H3N2)	A/Kanagawa/IC1870/2019	I38M	22.7	Yes	2019-01-24	356868	11
		A/Kanagawa/IC1817/2019	I38M/I mix (M: 62%)	24.5	Yes	2019-01-08	340692	9
		A/Kanagawa/IC18159/2019	I38M/R/I mix (M: 18%; R: 18%)	2.0	Yes	2019-02-21	2650503	7
		A/Kanagawa/AC1817/2018	I38M/T/I mix (M:21%; T: 40%)	88.0	Yes	2018-12-25	337453	8
		A/Mie/41/2018	I38T	368.0	No	2018-11-21	346647	12
		A/Yokohama/133/2018	I38T	118.9	Yes	2018-12-06	332908	6
		A/Yokohama/135/2018	I38T	75.4	Yes	2018-12-07	332910	7
		A/Kanagawa/IC1807/2018	I38T	67.2	Yes	2018-12-20	340687	14
		A/Yokohama/56/2019	I38T	224.1	Yes	2019-01-15	340695	1
		A/Kobe/18578/2019	I38T	164.2	Yes	2019-01-17	356751	4
		A/Wakayama/18/2019	I38T	75.9	Yes	2019-01-18	356870	14
		A/Kanagawa/IC1861/2019	I38T	117.0	Yes	2019-01-21	350486	14

		A/Kanagawa/IC1869/2019	I38T	252.1	Yes	2019-01-24	2650500	7
		A/Yokohama/87/2019	I38T	82.7	Yes	2019-01-25	341452	6
		A/Kanagawa/IC1894/2019	I38T	82.2	Yes	2019-01-29	2650501	13
		A/Kanagawa/IC18102/2019	I38T	61.6	Yes	2019-01-30	2650502	11
		A/Tokyo/18500/2019	I38T	91.1	No	2019-01-30	392484	86
		A/Aichi/118/2019	I38T	588.7	Yes	2019-01-31	391436	38
		A/Yokohama/88/2019	I38T	114.6	No	2019-01-31	341454	5
		A/Wakayama/23/2019	I38T	125.1	Yes	2019-02-05	356748	3
		A/Kanagawa/IC18141/2019	I38T	123.6	No	2019-02-07	345215	0
		A/Kanagawa/IC18143/2019	I38T	97.4	Yes	2019-02-07	364630	7
		A/Kanagawa/IC18144/2019	I38T	ND	Yes	2019-02-09	346656	10
		A/Hiroshima-C/30/2019	I38T	85.4	Yes	2019-02-11	363718	2
		A/Kanagawa/IC18160/2019	I38T	133.8	Yes	2019-02-21	2650504	10
		A/Kanagawa/AC1878/2019	I38T	84.4	No	2019-03-11	356753	6
		A/Kanagawa/AC1829/2019	I38T/I mix (T: 16%)	17.2	Yes	2019-01-15	340690	4
		A/Kanagawa/IC18103/2019	I38T/I mix (T: 66%)	5.3	Yes	2019-01-30	354788	8
		A/Wakayama/19/2019	I38T/I mix (T: 71%)	107.5	Yes	2019-01-25	356746	6
		A/Yokohama/99/2019	I38T/I mix (T: 82%)	60.6	Yes	2019-01-30	6915072	6
		A/Wakayama/22/2019	I38T/I mix (T: 83%)	29.7	Yes	2019-02-05	356747	2
		A/Kanagawa/IC1827/2019	I38T/I mix (T: 84%)	69.1	Yes	2019-01-12	337460	5
		A/Yokohama/61/2019	I38T/I mix (T: 88%)	239.4	Yes	2019-01-25	340699	4
2019/20	A(H1N1)pdm09	A/Kanagawa/AC1920/2019	E23K	7.4	No	2019-12-01	400629	10
2022/23	A(H1N1)pdm09	A/Mie/6/2023	I38T	ND	No	2023-08-25	18632811	3
	A(H3N2)	A/Kobe/22067/2022	Y24C	9.5	No	2022-12-12	17244516	1

		A/Yamagata/1/2022	Y24C	3.9	No	2022-12-13	17389726	19
		A/Shizuoka/3/2023	I38M	15.6	Yes	2023-01-12	17952502	12
		A/Kanagawa/IC2236/2023	I38M/T/I mix (M: 74%; T: 14%)	24.0	Yes	2023-04-11	17801734	65
		A/Yamagata/18/2023	I38T	161.6	No	2023-02-04	17789927	10
		A/Hiroshima/42/2023	I38T	67.9	No	2023-03-09	17801737	13
		A/Kanagawa/IC2235/2023	I38T	55.0	Yes	2023-04-06	17624324	44
		A/Miyazaki/68/2023	I38T	122.7	Yes	2023-05-19	18632791	16
		A/Miyazaki/73/2023	I38T	85.6	Yes	2023-05-20	18632797	39
		A/Kanagawa/IC2242/2023	I38T/I mix (T: 24%)	49.4	Yes	2023-07-04	18111778	14
		A/Nara/10/2023	E199G	3.6	No	2023-02-20	18246484	58
		A/Nara/12/2023	E199G	7.6	No	2023-02-21	17789922	37
		A/Nara/14/2023	E199G	4.0	No	2023-03-03	17995552	65
		A/Shizuoka/31/2023	E199K	5.0	No	2023-05-07	17995584	13
2023/24	A(H1N1)pdm09	A/Yamagata/103/2024	I38N	83.3	No	2024-03-12	19183931	14
		A/Yamagata/333/2023	I38T	199.4	No	2023-12-15	19045749	4
	A(H3N2)	A/Yokohama/163/2023	I38T	134.2	Yes	2023-10-10	19045774	8
		A/Kanagawa/IC2366/2024	I38T	344.1	Yes	2024-03-04	19018570	10
		A/Kanagawa/IC2321/2023	I38T/I mix (T: 62%)	194.0	Yes	2023-11-07	18632781	16

PA: polymerase acidic subunit; EC_{50}: 50% effective concentration; GISAID: Global Initiative on Sharing All Influenza Data; ND: not determined due to low virus titre.

The EC_{50} values of the viruses to baloxavir were determined by use of a focus reduction assay. In 2018/19, A(H1N1)pdm09 viruses were tested using MDCK cells, and A(H3N2) viruses using hCK cells. Since 2019/20, hCK cells have been used for all virus types.

[a] Fold-change in EC_{50} values for baloxavir compared with the median EC_{50} values of A(H1N1)pdm09 and A(H3N2) viruses collected in Japan during the same influenza season.

[b] Treatment with baloxavir before specimen collection.

Supplementary Table 3. Amino acid differences between influenza A(H3N2) viruses carrying the PA Y24C substitution and their most closely related wild-type viruses (n=4)

Isolate name	Amino acid substitution		EC_{50} (nM)
	PA 24	HA 9	
A/KOBE/22067/2022	C	S	39.50
A/YAMAGATA/1/2022	C	N	16.04
A/NIIGATA/68990/2022	Y	N	7.50
A/KANAGAWA/IC2209/2023	Y	N	8.14

PA: polymerase acidic protein; HA: haemagglutinin; EC_{50}: 50% effective concentration.

The EC_{50} values of the viruses to baloxavir were determined by use of a focus reduction assay.
